# The complete mitochondrial genome of *Aphis gossypii* Glover, 1877 (Hemiptera: Aphididae) collected in Korean peninsula

**DOI:** 10.1080/23802359.2019.1666051

**Published:** 2019-09-13

**Authors:** Jonghyun Park, Hong Xi, Yongsung Kim, Jongsun Park, Wonhoon Lee

**Affiliations:** a Infoboss Co., Ltd, Seoul, Republic of Korea;; b InfoBoss Research Center, Seoul, Republic of Korea;; c Department of Plant Medicine and Institute of Agriculture & Life Science, Gyeongsang National University, Jinju, Republic of Korea

**Keywords:** Mitochondrial genome, *Aphis gossypii*, Aphididae, intraspecies variations, Korea

## Abstract

*Aphid gosypii* Glover, 1877 is a widely recognized economically important aphid species in the world. We have determined mitochondrial genome of *A. gossypii* collected in Korean peninsula. The circular mitogenome of *A. gossypii* is 15,872 bp including 13 protein-coding genes, 2 ribosomal RNA genes, 22 transfer RNAs, and a single control region of 627 bp. The base composition was AT-biased (83.8%). In comparison of Chinese *A. gossypii* mitochondrial genomes with that of Korean sample, 61 single nucleotide polymorphisms and 3 insertions and deletions were identified, presenting lower level of those of *Nilaparvata lugens*, *Laodelphax striatellus*, and *Chilo suppresallis*.

*Aphis gossypii* Glover, 1877 is a cosmopolitan aphid species found from the temperate to the tropics. The species is notorious as agricultural pests not only for sucking sap from plants, but also a vector of more than 50 plant viruses spreading many viral diseases (Blackman and Eastop 2000). *Aphis gossypii* is extremely polyphagous, polymorphic, and can reproduce parthenogenetically (Zhang and Tong 1990), enabling it to feed on various crops (Cucurbitaceae, Rutaceae, and Malvaceae families; Schirmer et al. 2008) and multiply rapidly resulting difficult to control.

Here we present mitogenome collected in Seoul, Republic of Korea (37°45′74″N, 126°94′84″E; the specimen is stored in Gyeongsang National University, Korea, Accession number: Coll#WH0003). DNA was extracted using CTAB-based DNA extraction method manually (iNtRON biotechnology, INC., Korea). Raw sequences obtained from Illumina HiSeq2000 (Macrogen Inc., South Korea) were filtered by Trimmomatic 0.33 (Bolger et al. 2014) and *de novo* assembled by Velvet 1.2.10 (Zerbino and Birney 2008) and gaps including 548-bp-AT-rich region were closed with SOAPGapCloser 1.12 (Zhao et al. 2011), BWA 0.7.17, and SAMtools 1.9 (Li et al. 2009; Li 2013). Geneious R11 11.1.5 (Biomatters Ltd, Auckland, New Zealand) was used to annotate mitogenome based on that of Chinese *A. gossypii* (NC_024581; Zhang et al. 2016) with considering those of other aphid species.

*Aphis gossypii* mitogenome (Genbank accession is MN102349) is 15,872 bp long, which is longer than that of Chinese *A. gossypii* (NC_024581) by 3 bp. Its nucleotide composition is AT-biased (A + T is 83.8%) and contains 13 protein-coding genes, 2 rRNAs, and 22 tRNAs. The control region, presumably corresponding to single largest non-coding AT-rich region (627 bp, A + T is 84.5%), is same to that of Chinese *A. gossypii* (NC_024581).

In comparison of two *A. gossypii* mitogenomes, 61 single nucleotide polymorphisms (SNPs) and 3 insertions and deletions (INDELs) were identified. Three INDELs contribute 3-bp increase of length of Korean *A. gossypii* mitogenome. Level of intraspecies variations identified from samples between Korean and China is smaller than those of *Nilaparvata lugens* (Choi et al. 2019; Park, Kwon, et al. 2019), *Laodelphax striatellus* (Park, Jung, et al. 2019; Seo et al. 2019), and *Chilo suppresallis* (Park, Xi, et al. 2019).

We inferred the phylogenetic relationship of 35 mitogenomes, including two *A. gossypii* genomes and one outgroup species, *Bemisia tabaci* (Tay et al. 2016). Multiple sequence alignment was conducted by MAFFT 7.388 (Katoh and Standley 2013). Neighbor joining (10,000 bootstrap repeats) and maximum likelihood (1000 bootstrap repeats) phylogenetic trees were constructed using MEGA X (Kumar et al. 2018). Korean *A. gossypii* is clustered with Chinese *A. gossypii* (Figure 1), as expected, and all mitogenomes of Aphis genus form one clade, presenting monophyletic manner (Figure 1). In addition, it also presents that branch lengths of more than one mitogenome originated from *A. craccivora* and *Nurudea yanoniella* are similar to that of *A. gossypii* (Figure 1), indicating level of their intraspecies variations may be similar to each other. This mitogenome will be helpful to understand geographical intraspecies variations of *A. gossypii*.

**Figure 1.**
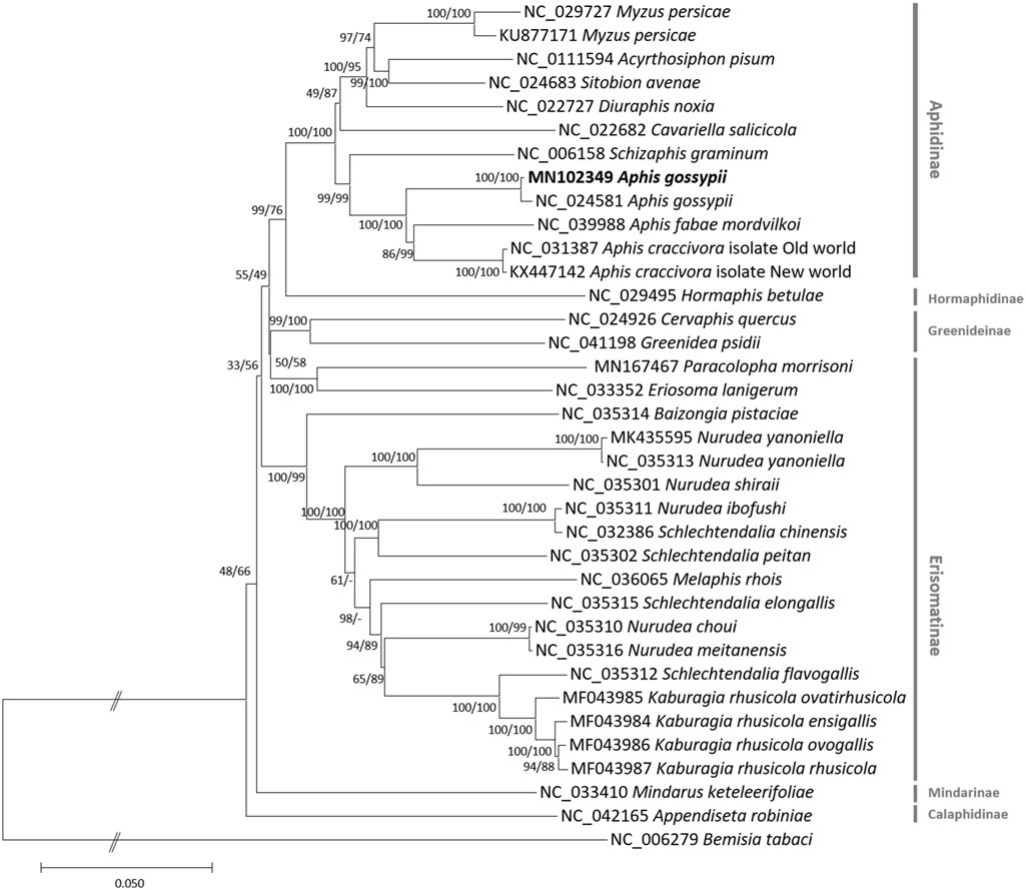
Neighbor joining (10,000 bootstrap repeats) and maximum likelihood (1,000 bootstrap repeats) phylogenetic trees of 35 mitochondrial genomes of Aphididae and one outgroup: two *Aphis gossypii* (MN102349 in this study and NC_024581), *Myzus persicae* (NC_029727, KU877171), *Acyrthosiphon pisum* (NC_011594), *Sitobion avenae* (NC_024683), *Diuraphis noxia* (NC_022727), *Cavariella salicicola* (NC_022682), *Schizaphis graminum* (NC_006158), *Aphis fabae mordvilkoi* (NC_039988), *Aphis craccivora* (NC_031387, KX447142), *Hormaphis Betula* (NC_029495), *Cervaphis quercus* (NC_024926), *Greenidea psidii* (NC_041198), *Eriosoma lanigerum* (NC_033352), *Paracolopha morrisoni* (MN167467), *Baizongia pistaciae* (NC_035314), *Nurudea yanoniella* (NC_035313 and MK435595), *Nurudea shiraii* (NC_035301), *Nurudea ibofushi* (NC_035311), *Schlechtendalia chinensis* (NC_032386), *Schlechtendalia peitan* (NC_035302), *Melaphis rhois* (NC_036065), *Schlechtendalia elongallis* (NC_035315), *Nurudea choui* (NC_035310), *Nurudea meitanensis* (NC_035316), *Schlechtendalia flavogallis* (NC_035312), *Kaburagia rhusicola ovatirhusicola* (MF043985), *Kaburagia rhusicola ensigallis* (MF043984), *Kaburagia rhusicola ovagallis* (MF043986), *Kaburagia rhusicola rhusicola* (MF043987), *Mindarus keteleerifoliae* (NC_033410), *Appendiseta robiniae* (NC_042165), and *Bemisia tabaci* (NC_006279) as an outgroup. Phylogenetic tree was drawn based on neighbour joining tree. The numbers above branches indicate bootstrap support values of neighbor joining and maximum likelihood phylogenetic trees, respectively.

## References

[CIT0001] Blackman RL, Eastop VF 2000 Aphids on the world's crops: an identification and information guide. 2nd ed. Chichester: John Wiley & Sons Ltd.

[CIT0002] Bolger AM, Lohse M, Usadel B 2014 Trimmomatic: a flexible trimmer for Illumina sequence data. Bioinformatics. 30:2114–2120. 24695404 10.1093/bioinformatics/btu170 PMC4103590

[CIT0003] Choi NJ, Lee B-C, Park J, Park J 2019 The complete mitochondrial genome of Nilaparvata lugens (Stål, 1854) captured in China (Hemiptera: Delphacidae): investigation of intraspecies variations between countries. Mitochondrial DNA Part B. 4:1677–1678.

[CIT0004] Katoh K, Standley DM 2013 MAFFT multiple sequence alignment software version 7: improvements in performance and usability. Mol Biol Evol. 30:772–780. 23329690 10.1093/molbev/mst010 PMC3603318

[CIT0005] Kumar S, Stecher G, Li M, Knyaz C, Tamura K 2018 MEGA X: molecular evolutionary genetics analysis across computing platforms. Mol Biol Evol. 35:1547–1549. 29722887 10.1093/molbev/msy096 PMC5967553

[CIT0006] Li H 2013. Aligning sequence reads, clone sequences and assembly contigs with BWA-MEM. arXiv preprint arXiv:13033997.

[CIT0007] Li H, Handsaker B, Wysoker A, Fennell T, Ruan J, Homer N, Marth G, Abecasis G, Durbin R 2009 The sequence alignment/map format and SAMtools. Bioinformatics. 25:2078–2079. 19505943 10.1093/bioinformatics/btp352 PMC2723002

[CIT0008] Park J, Jung JK, Ho Koh Y, Park J, Seo BY 2019 The complete mitochondrial genome of *Laodelphax striatellus* (Fallén, 1826) (Hemiptera: Delphacidae) collected in a mid-Western part of Korean peninsula. Mitochondrial DNA Part B. 4:2229–2230. 10.1080/23802359.2019.1623112 PMC7687552 33365487

[CIT0009] Park J, Kwon W, Park J, Kim H-J, Lee B-C, Kim Y, Choi NJ 2019 The complete mitochondrial genome of *Nilaparvata lugens* (stål, 1854) captured in Korea (Hemiptera: Delphacidae). Mitochondrial DNA Part B. 4:1674–1676.

[CIT0010] Park J, Xi H, Kwon W, Park C-G, Lee W 2019 The complete mitochondrial genome sequence of Korean *Chilo suppressalis* (Walker, 1863)(Lepidoptera: Crambidae). Mitochondrial DNA Part B. 4:850–851.

[CIT0011] Schirmer S, Sengonca C, Blaeser P 2008 Influence of abiotic factors on some biological and ecological characteristics of the aphid parasitoid *Aphelinus asychis* (Hymenoptera: Aphelinidae) parasitizing *Aphis gossypii* (Stenorrhyncha: Aphididae). Eur J Entomol. 105:121–129.

[CIT0012] Seo BY, Jung JK, Ho Koh Y, Park J 2019 The complete mitochondrial genome of *Laodelphax striatellus* (Fallén, 1826) (Hemiptera: Delphacidae) collected in a southern part of Korean peninsula. Mitochondrial DNA Part B. 4:2242–2243. 10.1080/23802359.2019.1624645 PMC7687393 33365493

[CIT0013] Tay W, Elfekih S, Court L, Gordon K, De Barro P 2016 Complete mitochondrial DNA genome of *Bemisia tabaci* cryptic pest species complex Asia I (Hemiptera: Aleyrodidae). Mitochondrial DNA Part A. 27:972–973. 10.3109/19401736.2014.926511 24960562

[CIT0014] Zerbino DR, Birney E 2008 Velvet: algorithms for *de novo* short read assembly using de Bruijn graphs. Genome esearch. 18:821–829. 10.1101/gr.074492.107 PMC2336801 18349386

[CIT0015] Zhang GX, Zhong TS 1990 Experimental studies in some aphid life-cycle patterns and the hybridization of two sibling species In: Campbell RK and Eikenbary RD, editors. Aphid–plant genotype interactions. New York: Elsevier; p. 37–50.

[CIT0016] Zhang S, Luo J, Wang C, Lv L, Li C, Jiang W, Cui J, Rajput LB 2016 Complete mitochondrial genome of *Aphis gossypii* Glover (Hemiptera: Aphididae). Mitochondrial DNA Part A. 27:854–855. 10.3109/19401736.2014.919474 24865902

[CIT0017] Zhao QY, Wang Y, Kong YM, Luo D, Li X, Hao P 2011 Optimizing *de novo* transcriptome assembly from short-read RNA-Seq data: a comparative study. BMC Bioinformatics. 12:S2. 10.1186/1471-2105-12-S14-S2 PMC3287467 22373417

